# Natural hypothalamic circuit dynamics underlying object memorization

**DOI:** 10.1038/s41467-019-10484-7

**Published:** 2019-06-07

**Authors:** Christin Kosse, Denis Burdakov

**Affiliations:** 1 The Francis Crick Institute, London, NW1 1AT UK; 2 Laboratory of Molecular Genetics, The Rockefeller University, New York, NY 10065 USA; 3 Neurobehavioural Dynamics Lab, Institute for Neuroscience, D-HEST, Swiss Federal Institute of Technology / ETH Zürich, Zürich, 8603 Switzerland

**Keywords:** Neuroscience, Neurophysiology

## Abstract

Brain signals that govern memory formation remain incompletely identified. The hypothalamus is implicated in memory disorders, but how its rapidly changing activity shapes memorization is unknown. During encounters with objects, hypothalamic melanin-concentrating hormone (MCH) neurons emit brief signals that reflect object novelty. Here we show that targeted optogenetic silencing of these signals, performed selectively during the initial object encounters (i.e. memory acquisition), prevents future recognition of the objects. We identify an upstream inhibitory microcircuit from hypothalamic GAD65 neurons to MCH neurons, which constrains the memory-promoting MCH cell bursts. Finally, we demonstrate that silencing the GAD65 cells during object memory acquisition improves future object recognition through MCH-receptor-dependent pathways. These results provide causal evidence that object-associated signals in genetically distinct but interconnected hypothalamic neurons differentially control whether the brain forms object memories. This gating of memory formation by hypothalamic activity establishes appropriate behavioral responses to novel and familiar objects.

## Abstract (web summary)

Hypothalamus is implicated in memory disorders but the neural mechanisms are unknown. Here, the authors report that MCH expressing hypothalamic neurons respond to novel object exposure, are inhibited by local GAD65 expressing neurons and these local circuit interactions are causally involved in object memory formation.

## Introduction

The ability to memorize objects enables one to react differently to novel and familiar objects. This ability is fundamental for normal life and impaired in many common and devastating brain pathologies^1,2^. It is still debated which brain structures and signals are critical for the formation of object memory^3,4^. Intense research into this topic traditionally focused on brain areas such as the perirhinal cortex and hippocampus^2,4^. In contrast, causal roles of neural dynamics of the hypothalamus have been underexplored, despite over half a century of evidence implicating this region in memory disorders^5–9^. The hypothalamus contains multiple neuronal types interconnected in complex and poorly-understood ways, including neurons expressing the peptide neurotransmitter melanin-concentrating hormone (MCH)^10^ that innervate many brain areas thought to be important for memory control^8,11^. While originally MCH_LH_ neurons were only thought to be active during sleep^12^, it was found recently that they are also active during awake spatial exploration^13^. However, it remains unknown whether this natural MCH_LH_ cell activity during wakefulness influences object memory formation, because wakefulness-specific silencing of MCH_LH_ neurons in the context of object memorization has not been performed. The neural circuits shaping MCH_LH_ cell activity during wakefulness and roles of such circuits in memory formation also remain undefined. Here we explored these unknowns by reversible silencing of MCH_LH_ cells and their upstream neurons (newly identified here) during encounters with objects, combined with examining the subsequent behavioral reactions to novel and previously encountered objects.

## Results

### Natural MCH cell activity represents object novelty

**Fig. 1:**
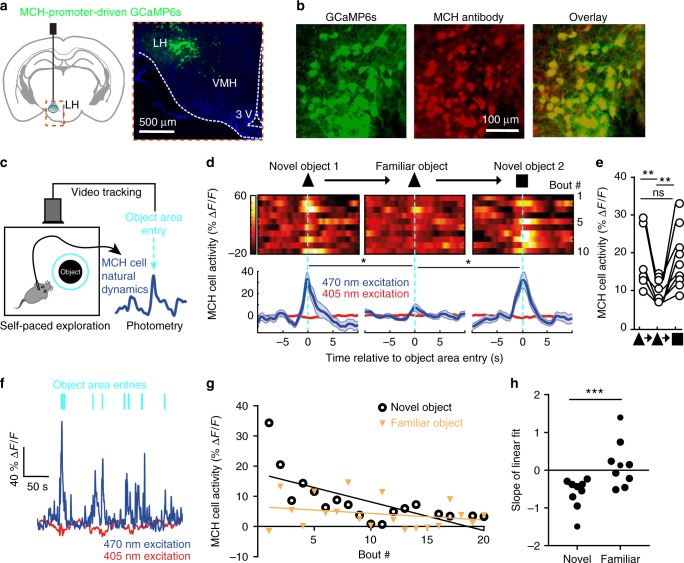
Representations of novel objects in MCH cell dynamics. **a** Targeting scheme (left) and expression (right) of GCaMP6s in MCH_LH_ cells. **b** Confirmation of GCaMP6s expression in MCH_LH_ cells (see “Methods”). **c** Schematic (left) of MCH_LH_ cell recording concurrent with behavioral tracking. **d** Top, representative heatmaps of MCH_LH_::GCaMP6s fluorescence (at 470 nm excitation) aligned to object-area entry. First 10 object area entries from one mouse (representative data of *n* = 9 mice). Bottom, group data (means ± s.e.m of *n* = 10 objects visits for one mouse), also showing negative control from 405 nm excitation. One-way RM ANOVA F(2,27) = 5.505, *p* = 0.0099, Tukey’s multiple comparisons: Novel object 1(triangle) vs novel object: *p = 0.0227, Novel object vs. novel object 2 (rectangle): *p = 0.0189. **e** Quantification of peak MCH_LH_ cell activity from D, n = 9 mice. One-way RM ANOVA *F*(1.694, 13.55) = 16.52, *p* = 0.0004, Tukey’s multiple comparisons post-tests: novel object 1 (triangle symbol) vs novel object 2 (square symbol) *p* = 0.9991 (ns), novel object 1 vs familiar object (same object presented 30 min later) **p = 0.0021, familiar object (middle triangle symbol) vs novel object 2 (square symbol): **p = 0.0073. **f** Representative MCH_LH_ cell responses from one mouse to a sequence of self-paced novel object area entries. **g** Representative peak MCH_LH_ cell responses from one mouse to a self-paced sequence of novel (NO) and familiar (FO) object area entries; straight lines are linear fits to the data. **h** Quantification of data in G for *n* = 9 mice, paired t-test: ***p = 0.0074, *t*(8) = 3.556

Recordings of natural MCH_LH_ cell activity during self-paced navigation in object-containing arenas revealed that MCH_LH_ cells emitted activity bursts when mice encountered objects (Fig. 1a–f, Supplementary Fig. 1A–E; encounter was defined by real-time video-tracking as an entry of mouse nose into the object area, see “Methods”). Such object-related activity was not observed in LH hypocretin/orexin neurons (Supplementary Fig. 1F), indicating a cell-type-specificity of LH responses to object encounters. When recorded continuously during sequential presentation of novel and familiar objects to the same mice, the novel-object-encounter-associated MCH_LH_ signals decreased as mice spent more time with the object (Fig. 1d–h; Supplementary Fig. 1E), but increased again when they were presented with a new novel object (Fig. 1d, e; Supplementary Fig. 1C). When mice were presented with familiar objects, the object-encounter-associated MCH_LH_ signals during consecutive object encounters tended to remain small in amplitude, in contrast to novel-object-associated signals that were initially large and decayed during consecutive object encounters (Fig. 1f–h). This object familiarization-evoked reduction in MCH_LH_ signals persisted when the familiar object was moved to a new location (Supplementary Fig. 1B, D), and was maintained for up to 20 h (Supplementary Fig. 1C, D). Together, these properties of object-encounter-associated MCH_LH_ signals are consistent with signals associated with object memorization.

### Causal link of natural MCH cell dynamics and object memory

**Fig. 2:**
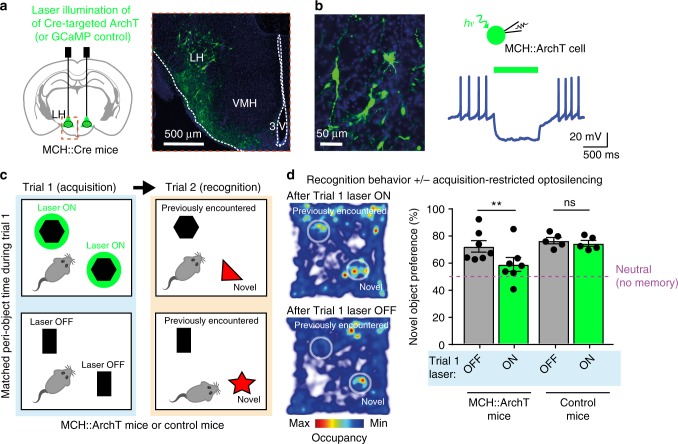
Natural MCH cell bursts gate object memory formation. **a** Targeting scheme (left) and expression (right) of ArchT in MCH_LH_ cells. **b** Patch-clamp recording (right) confirming silencing of MCH_LH_::ArchT-YFP cells (left) by green light (*n* = 5 cells). **c** Experimental scheme: self-paced exploration of two identical novel objects for the same cumulative peri-object time (trial 1) followed by quantifying exploration of the same arena with one of the previously encountered objects replaced by a novel object (trial 2). **d** Left: sample heatmaps showing relative time spent with objects on trial 2. Right: group data (individual points and their means ± s.e.m). Object-area-entry-coupled bilateral LH laser illumination during trial 1 reduced object recognition in trial 2 in MCH_LH_::ArchT mice (*n* = 7) but not in control (MCH_LH_::GCaMP) mice (*n* = 5) (2-way ANOVA: *F*(1, 8) = 7.43, *p* = 0.0260, Sidak’s multiple comparisons test: **p = 0.0034, ns = *p* = 0.7073). In MCH_LH_::ArchT mice, trial 1 laser ON group, the preference for the novel object during trial 2 was not different from “no memory” (neutral, 50%) criterion (one sample t-test against 50% preference: *t*(6) = 1.775, *p* = 0.1262), whereas in all other groups significant preference was seen (one sample t-tests against 50% preference: MCH_LH_::ArchT mice laser OFF, *t*(6) = 5.276, *p* = 0.0019; control mice laser OFF, t(4) = 11.37, *p* = 0.0003; control mice laser ON, t(4) = 11.42, *p* = 0.0003)

To probe whether the object encounter-associated signals of MCH_LH_ cells play a causal role in object memory formation, we close-looped real-time video-tracking of object encounters to MCH_LH_ cell optosilencing in MCH_LH_::ArchT mice (Fig. 2a–c, see “Methods”). We did this in the context of a classic object memory test^14,15^, where mice are exposed to pairs of objects in 2 temporally separated trials (Fig. 2c). In trial 1 (memory acquisition phase) they encountered two identical novel objects, with or without the object-associated MCH_LH_ cell optosilencing (Fig. 2c). In trial 2 (object recognition test), which involved no optosilencing, object memory was quantified as time spent with a novel vs. the previously encountered object (Fig. 2c, this quantifies object memory since in this test mice are normally less drawn to previously encountered objects^14,15^). To prevent variations in sensory or laser exposure from affecting memorization, we tracked the total object encounter time in trial 1, and matched its value across compared conditions (see “Methods” section “Object recognition tests with controlled familiarization time”). MCH_LH_ cell optosilencing selectively during object encounters in trial 1 prevented MCH::ArchT mice from recognizing the previously encountered objects in trial 2 (Fig. 2d). In contrast, trial 2 object recognition was normal when the same closed-loop LH laser illumination experiment was performed in control mice lacking the optoinhibitory opsin (MCH_LH_::GCaMP mice) (Fig. 2d). This shows that the natural MCH_LH_ cell activity during initial object encounters is necessary for the object to be treated as familiar in the future, i.e., for object recognition memory formation. The controlled design of these experiments (exposure time matching, mixed order within-mouse repeats, control mice, see “Methods”) indicates that the disruption of object memory formation by the temporally-targeted optosilencing of MCH_LH_ cells was not due to differences in sensory exposure, or order or laser-related effects.

### Identification of inhibitory GAD65 → MCH neural microcircuit

**Fig. 3:**
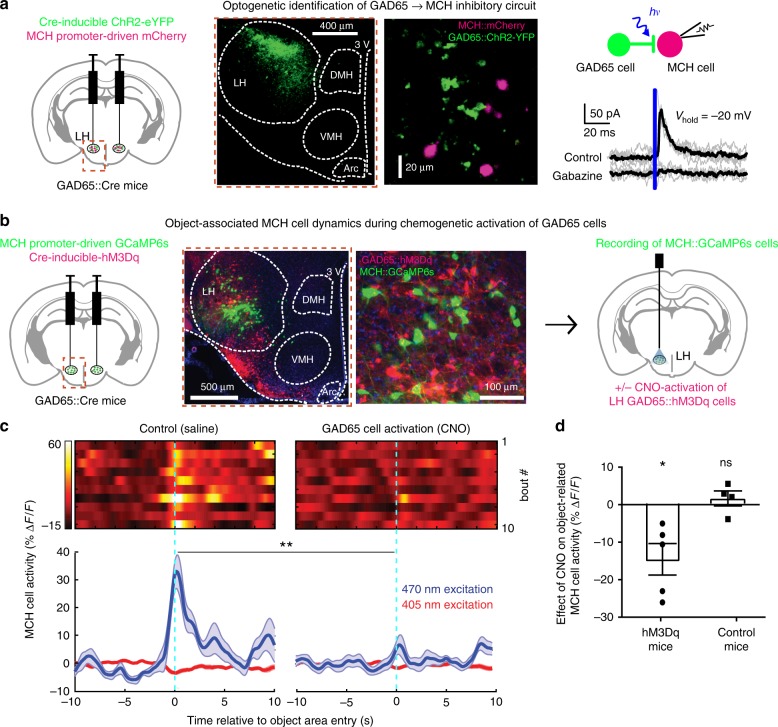
Identification of GAD65→MCH inhibitory microcircuit. **a** Targeting schematic (left) for expression of ChR2 in GAD65_LH_ cells and mCherry in MCH_LH_ cells (middle panels). Right, GAD65_LH_::ChR2 optostimulation evokes a gabazine–sensitive current in MCH_LH_::mCherry cells. Gray lines are individual trials, black lines are trial averages; *n* = 14/16 cells were connected; latency between GAD65_LH_ cell optostimulation and the onset of postsynaptic current: 0.83 ± 0.8 ms (*n* = 14 cells), synaptic current size is quantified in Supplementary Fig. 3C). **b** Targeting schematic (left) and expression (two middle panels) of GCaMP6s in MCH_LH_ cells and hM3Dq in GAD65_LH_ cells, for recording of MCH_LH_::GCaMP6s cell activity during GAD65_LH_::hM3Dq cell modulation (right). **c** Top, representative heatmaps of MCH_LH_::GCaMP6s fluorescence (at 470 nm excitation) aligned to object-area entry. First 10 object area entries from one mouse (representative data of *n* = 5 mice). Bottom, corresponding group data (means ± s.e.m of *n* = 10 visits), also showing negative control from 405 nm excitation. Chemogenetic activation of GAD65_LH_::hM3Dq cells decreased object-area-entry-associated MCH_LH_ cell activity peaks (*t*(18) = 3.805, **p = 0.0013, unpaired *t*-test, representative data comparing 10 object encounters before and after CNO from one mouse, group data are given in **d**). The late activity at around 5 and 10 s reflect activity outside the object area, which was not investigated further. **d** Group data (individual points and means ± s.e.m), showing effect of CNO (i.e., response in CNO minus response in saline) on peak peri-object MCH_LH_ cell signals in negative control mice (MCH_LH_::GCaMP6s, *n* = 4), and in hM3Dq mice (MCH_LH_::GCaMP6s and GAD65_LH_::hM3Dq, *n* = 5); *p = 0.0257, *t*(4) = 3.466, one-sample *t*-test, *n* = 5 mice; ns = *p* = 0.4623, *t*(3) = 0.8406, one-sample *t*-test, *n* = 4 mice

The above findings show that inhibition of the object-associated MCH_LH_ activity selectively during memory acquisition is a powerful way to control object memory formation. In search for neural origins of this inhibition, we used channelrhodopsin (ChR2)-assisted circuit mapping to probe functional interactions of MCH_LH_ and neighboring non-MCH cells with local GAD65_LH_ neurons, a recently-characterized LH neural type whose downstream cell targets are yet unknown^16^. In mouse brain slices, optostimulation of GAD65_LH_::ChR2 cells evoked rapid GABAergic inhibitory input in MCH_LH_ cells (Fig. 3a) but not in the neighboring LH orexin/hypocretin cells (Supplementary Fig. 3B). Optostimulation of MCH_LH_::ChR2 cells did not evoke detectable input in GAD65_LH_ cells (Supplementary Fig. 3A), suggesting a unidirectional GAD65 → MCH LH microcircuit. In complementary in vivo circuit-connectivity screens in object-exploring mice, chemogenetic activation of GAD65_LH_::hM3Dq cells (Fig. 3b, see “Methods”) was able to suppress the novel object encounter-associated activity MCH_LH_ cell bursts (Fig. 3c, d). Thus, a functional inhibitory GAD65 → MCH LH circuit exists that is sufficiently powerful to suppress object-encounter-associated MCH_LH_ cell activity.

### Natural GAD65 cell activity controls memory via MCH pathways

**Fig. 4:**
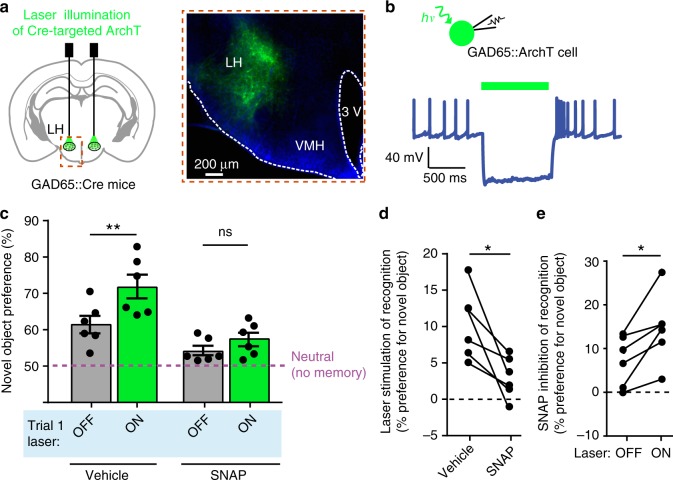
GAD65_LH_ cells control object memory via MCH signaling. **a** Targeting schematic (left) and expression (right) of ArchT in GAD65_LH_ cells. **b** Patch-clamp recording from GAD65_LH_::ArchT cells confirming cell inhibition by green light (*n* = 5 cells). **c** Role of GAD65_LH_ cell activity in object memory formation (experimental design as in Fig. 2c). Data are shown as individual points (mice) and their means ± s.e.m. Object-area-entry-associated GAD65_LH_ cell optosilencing during trial  1 increased object recognition in trial 2 in the absence (2-way ANOVA, *F*(1, 5) = 17.1, *p* = 0.0090, Tukey’s multiple comparisons test: **p = 0.0036) but not presence of MCHR blocker SNAP94847 (SNAP) (2-way ANOVA, *F*(1, 5) = 17.1, *p* = 0.0090, Tukey’s multiple comparisons test: ns = *p* = 0.2931), *n* = 6 mice. **d** Quantification of trial 2 object recognition enhancement by the trial 1 GAD65_LH_ cell optosilencing, in the presence and absence of SNAP (*n* = 6 mice, paired *t*-test: *t*(5) = 3.488, *p = 0.0175). **e** Quantification of trial 2 object recognition enhancement by the trial 1 SNAP, with and without concurrent GAD65_LH_ cell optosilencing, *n* = 6 mice, paired *t*-test: *t*(5) = 3.488, *p = 0.0175

To investigate whether the natural GAD65_LH_ cell activity influences object memory acquisition via the MCH system, we repeated the memory acquisition—coupled optogenetic interference (Fig. 2) with GAD65_LH_ optosilencing. The GAD65_LH_ cell optosilencing targeted to object encounters during object memory acquisition significantly increased subsequent novel object preference during object recognition (Fig. 4c, d). This indicates that the natural activity of GAD65_LH_ cells opposes object memory formation, as expected from the inhibitory GAD65 → MCH LH circuit. If this effect of GAD65_LH_ cells on object memory formation is mediated by the inhibitory GAD65 → MCH LH circuit, then it should be diminished by blocking MCH cell outputs. Consistent with this prediction, when the GAD65_LH_ cell optosilencing was performed concurrently with MCH receptor blockade using the MCH receptor antagonist SNAP94847 (20 mg/kg i.p., see “Methods”), the effect of the GAD65_LH_ cell optosilencing was abolished (Fig. 4c, d). Conversely, object memory formation driven by natural MCH signaling (isolated by quantifying behavior with and without SNAP94847 in individual mice) was significantly increased by GAD65_LH_ cell inhibition (Fig. 4e), confirming that MCH_LH_ cells regulate behavior according to GAD65_LH_ cell tone. This shows that the object-encounter-associated, natural GAD65_LH_ cell activity governs object memory formation via MCH-receptor-dependent pathways.

## Discussion

Our findings indicate that mice do not recognize objects unless their MCH cells are active during the prior object encounters, and that a novel GAD65 → MCH microcircuit governs the size of the memory-controlling MCH cell bursts. The type of object recognition memory that we studied is fundamentally important for normal behavior in both humans and animals^2,17^. To the best of our knowledge, our study is the first to make causal links between the transient, object-associated dynamics of hypothalamic neurons and memory formation. Previous molecular and pharmacological studies linked MCH peptide signaling to avoidance memory^8,9,18,19^, but did not involve temporally precise manipulation of ongoing MCH cell activity at behaviorally-relevant timescales, and thus contained no indication when the natural MCH cell activity influences memory, nor how upstream circuits shape these memory-gating MCH signals. Our data thus provide causal evidence for the role of object-associated signals in a hitherto unknown hypothalamic circuit in object recognition memory formation.

These results support broader roles of rapid hypothalamic signals in cognition than previously considered, as also supported by recent data on other hypothalamic neurons^20–23^. Furthermore, our data suggest that the hypothalamus not only relays a critical input for computation of appropriate behavior, but that local hypothalamic microcircuits also contribute to this computation. This contribution can be critical for fundamentally important behavior, since disrupting natural LH processing transiently and specifically during initial object encounters prevented mice from displaying normal behavioral responses (i.e., recognition) to novel and familiar objects in the future (Figs. 2 and 4). While our study focussed on inanimate objects in order to isolate object memory effects from reward or social motivators, in the future it will be interesting to investigate how local hypothalamic processing affects novelty and familiarity behavior towards more complex objects such as food and conspecifics.

Our finding that GAD65 → MCH LH circuit is important for object memory does not rule out that this circuit may also be involved in other functions. While so far we found no evidence that MCH_LH_ or GAD65_LH_ cells are involved in spatial working memory (Supplementary Fig. 4), nor that MCH cells signal spatial locations (Supplementary Fig. 1B, D), we did observe that they may signal novel sensory qualities of food (Supplementary Fig. 5). The possibility that this circuit may signal multiple novel sensory experiences does not undermine the validity and importance of our findings relating to its involvement in object recognition memory. At the neuroanatomical level, MCH_LH_ cell axons and MCH receptors are found brain-wide, including multiple regions speculated to be involved in object memory^3,4,9,11,24^, where MCH is proposed to alter synaptic plasticity thus making memories more likely to form^8,9^. Probing this broader downstream connectivity of the GAD65 → MCH LH circuit will improve our understanding of hypothalamic gating of cognition, though this is likely to be challenging given the breadth of MCH_LH_ projections and the current lack of consensus about relative roles played by different brain regions in object recognition memory. Upstream, it would be interesting to probe whether known regulatory inputs to MCH_LH_ and GAD65_LH_ cells—for example orexin, insulin, and glucose^13,16,25,26^—may act to match memory-related processes to stress and energy levels.

In summary, our study identifies a neural circuit that governs brain representations of object novelty, and links the natural object-related activity of this circuit to a vital cognitive function: object recognition memory formation. This circuit mechanism for the control of object memory formation offers new insights into neuromodulation of valued cognitive abilities that are key targets of rehabilitation in neuropsychiatric disease.

## Methods

### Genetic targeting

All procedures followed United Kingdom Home Office regulations and were approved by the Animal Welfare and Ethical Review Panel of the Francis Crick Institute. Mice were kept on a standard 12-h/12-h light/dark cycle and on standard mouse chow and water ad libitum. Adult male and female mice (at least 8-week old) were used for in vitro experiments. Adult male mice were used for behavioral experiments, which were performed during the dark phase. The following previously characterized and validated transgenic mouse lines (or their crosses) were used, where indicated: MCH::Cre mice^27^, GAD65::Cre mice^28^, GAD65::GFP mice^29^, orexin::GFP mice^30^. The GAD65::Cre mice were bred in homozygous (hom)-WT pairs with C57BL/6 mice; all other transgenic mice were bred in het-WT pairs with C57BL/6 mice. For brain surgeries, mice were anesthetized with isoflurane and injected with meloxicam (2 mg/kg of body weight, s.c.) for analgesia. After placement into a stereotaxic frame (David Kopf Instruments), a craniotomy was performed and a borosilicate glass pipette was used to inject viral vectors bilaterally into the LH. Two injections (each 75 nL) were made into the LH in each hemisphere (bregma: −1.30 mm, midline: ±1 mm, from brain surface: 5.20 mm and 5.25 mm). Before any manipulations, mice were allowed to recover from surgery for at least 1 week while single-housed. To target expression of the activity indicator GCaMP6s to MCH_LH_ neurons, we used an AAV vector carrying the 0.9 kb preproMCH gene promoter^31^, AAV9.pMCH.GCaMP6s.hGH (1.78 × 10^14^ gc/mL; Vigene Biosciences). The specificity of GCaMP6s expression was confirmed by staining with MCH antibody (Fig. 1b, 93% specificity was observed by analysis of MCH immunoreactivity colocalisation in 426 GCaMP6s neurons from three brains). For optogenetic silencing of MCH_LH_ or GAD65_LH_ neurons, we injected Cre-dependent AAV8.Flex-ArchT-GFP (4.6 × 10^12^ gc/ml; UNC Vector Core) into LH of the MCH::Cre or GAD65::Cre mice, respectively. For ChR-assisted circuit mapping, “FLEX switch” ChR2 constructs were injected into LH of the MCH::Cre or GAD65::Cre mice, as indicated. These constructs were either AAV1.EF1.flox.hChR2(H134R)-mCherry.WPRE.hGH (8.78 × 10^12^ gc/mL; UPenn Vector Core) or AAV1.EF1.DIO.hChR2(H134R)-YFP.WPRE.hGH (6.2 × 10^12^ gc/mL; UPenn Vector Core). Cre-dependent “DREADD” chemogenetic actuator hM3Dq was targeted to GAD65_LH_ neurons in GAD65::Cre mice by injecting the vector AAV8.hSvn-DIO-hm3D(Gq)-mCherry (2.2 × 10^12^ genome copies (gc)/mL; UNC Vector Core) into LH of the GAD65::Cre mice^16^.

### Fiber photometry

After LH injection of MCH-promoter-driven GCaMP6s, alone in C57/Bl6 mice or in combination with the Cre-dependent activatory DREADD (hM3Dq) in GAD65::Cre mice, fiberoptic implants were stereotaxically installed with the fiber tip above the LH (1.35 mm caudal from bregma, 1.0 mm lateral from midline, and 5 mm ventral from brain surface) and fixed to the skull as in our previous work^32,13^. This method is estimated to capture fluorescence signals from within ≈500 μm of the fiber tip^32^. Fiber tip locations were verified in each mouse by examining slices with a visible fiber tract. During fiber photometry experiments^32^, the excitation mode was set to provide interleaved 405 nm and 470 nm excitation light pulses via LEDs^33^. Fluorescence emission produced by 405 nm excitation is not sensitive to calcium and thus provides a real-time control for motion artefacts^33^. Fluorescence signals were normalized to produce the plotted % Δ*F*/*F* values as follows: Δ*F*/*F* = 100 * (Fr − *F*)/*F*, where Fr is the raw signal and *F* is the mean of the first 10 s of trial. Before photometry recordings, mice were habituated to the recording chamber, the plugging in procedure, and (where relevant) i.p. injections. On the day of fiber photometry recordings, mice were given 10 min to adjust to the chamber before an object was introduced. During the next 1 h mice had time to familiarize themselves with the object, after which the object was removed (i.p. injections of CNO or saline where performed where relevant at this point). Thirty minutes after this, the familiar object was reintroduced and mice had free access to explore it while their brain signals were recorded, and head location was video-tracked (Ethovision XP, 15 frames/s). The novel object trial followed, by exchanging the familiar object for a novel object and allowing mice to freely explore the novel object while their brain signals were recorded, and head location was video-tracked. Object exploration bouts were detected by nose video-tracking (Ethovision XP), and their onset defined as the first frame when the mouse nose entered the object area (defined as a 3 cm-wide perimeter around the object). Choice of objects for familiar and novel trials were based on a crossover design to avoid any confounding factors due to differences in objects. To compare MCH::GCaMP6s_LH_ calcium signals between mice, we selected the first 10 exploration bouts of each mouse for the novel and familiar objects, and used these data to derive averaged signal per mouse. During the first 10 entries the object was investigated more frequently if it was novel (Supplementary Fig. 2H), and since our aim was to define neural correlates of behavioral responses to novel objects, we chose the first 10 entries for analysis of MCH photometry signals.

### Closed-loop neural optosilencing

Mice were bilaterally LH-injected with Cre-dependent ArchT (or, in control experiments, Cre-dependent GCaMP6s), and bilaterally implanted with intra-LH optical fibers using the coordinates and procedures as described above for fiber photometry. Three weeks after surgery, mice were handled and habituated to the recording arena before any procedure started. For experiments, a green laser (532 nm, LaserGlow) was connected to the bilateral fibre implants to yield ≈20 mW light power output at the fiber tip. Since photometry recordings showed an onset of increased MCH neuron activity before mice entered the object area (Fig. 1d), we paired bilateral LH laser illumination with times when mouse nose was <2 cm away from object area (i.e., within 5 cm perimeter from object). For control experiments investigating the effect of silencing GAD65_LH_ or MCH_LH_ neurons on object exploration, mice were freely behaving for 10 min in an open field arena with two identical objects, and the peri-object area of one object (defined as above) was paired with the bilateral LH laser illumination to test for the GAD65_LH_ or MCH_LH_ neuron effects on object exploration (Supplementary Fig. 2A–D). The propensities for self-paced object investigation of GAD65::Cre and MCH::Cre mice were investigated in control experiments and found to be similar (Supplementary Fig. 2I).

### Object recognition memory tests

For object recognition memory tests^15^ (Figs. 2c, d;  4c–e), during the laser ON familiarisation, the bilateral LH laser illumination was triggered whenever the mouse entered the peri-object area (as defined above) of either object. No laser was applied during recognition trials. Laser OFF familiarisation was performed in the same mice with the same temporal contingencies as laser ON familiarisation, but with a new set of objects. After 1 h of retention interval, during which mice were returned to their home cages and no experimental manipulations were performed, the recognition trial (= trial 2) consisted of 10 min during which mice freely explored one object from the previous familiarisation trial (familiar object) and one novel object. Sets of novel and familiar objects were alternated between mice in a crossover design. For novel object recognition tests where MCH receptors were blocked with SNAP 94847 (Fig. 4c–e), mice were i.p. injected with SNAP or vehicle solutions 45 min before trial 1. In these experiments, a longer interval between familiarisation/acquisition and recognition trials was used (20 h), to ensure that MCH receptors were only blocked during memory acquisition, and that mice were unimpaired by SNAP during recognition tests.

Our aim was to specifically examine the effects of LH optosilencing on memory formation, independently of factors such as the duration of sensory exposure to objects during familiarisation/memorisation. Therefore, in laser ON and laser OFF familiarization trials, a constant cumulative exposure of mice to objects was imposed, by real-time video tracking of the cumulative object encounter time (time when the mouse nose was in the object area), and terminating all trials when the same cumulative object encounter time (30 s) was reached. This ensured that differences in object memory acquisition were not due to variation in initial object exposure between different mice or trials, or different optosilencing conditions that may otherwise have influenced the total object investigation time as suggested by our control experiments (Supplementary Fig. 2A–D).

### Y maze test of spatial memory

Continuous spontaneous alterations in a Y maze were measured with and without concurrent optogenetic silencing in the same mice (sequence of optogenetic silencing and laser off was alternated between mice) (Supplementary Fig. 4). Mice were connected to bilateral patch cords 10 min before start of the experiments and then transferred to the center of a standard Y maze (3 arms, 30 cm long, 120° apart). During the following 8 min, mice were free to explore the arms of the Y maze whilst video tracking with Noldus Ethovision scored the spontaneous alterations defined as consecutive entries into three different arms^34,35^.

### Experimental sequences in behavioral experiments

Crossover-like experimental designs were used in all in vivo photometry and optogenetic experiments, to prevent artefacts and biases and isolate the effects of variables under investigation. Specifically, presentations of novel and familiar object were alternated within and between mice to avoid behavioral fatigue or order effects. Photometry experiments were designed to expose the same mouse to sequences of novel and familiar objects that avoided behavioral habituation or calcium indicator degradation as confounding factors (e.g., novel → familiar → novel, Fig. 1d, Supplementary Fig. 1B, C). Optogenetic experiments were based on a crossover-like design where manipulations involving drugs, laser light, or mouse genotype were arranged in a Latin square to avoid any confounding factors due to day to day differences or carry-over effects. To prevent potential arena side biases from influencing the results of experiments involving two objects positioned at different sides of arena, trials were repeated with laser OFF and ON sides reversed; the presented results are an average of both trials.

### Channelrhodopsin-assisted circuit mapping in brain slices

For brain slice patch-clamp recordings combined with optogenetics^16,36^, LH slices were prepared at least 2 months after virus injection. Coronal brain slices containing the LH were cut at 250 μm thickness while immersed in ice-cold slicing solution. Slices were incubated for 1 h in artificial cerebrospinal fluid (ACSF) at 35 °C, and then transferred to a submerged-type recording chamber. Neurons containing fluorescent markers were visualized with an Olympus BX61WI microscope with an oblique condenser and fluorescence filters. Excitation light was delivered from a LAMBDA DG-5 beam switcher (Sutter) with a xenon lamp and ET470/40 (for ChR2) or ET500/20 (for ArchT) bandpass filters. A 40× 0.8NA objective was used to deliver pulses of excitation light (∼10 mW/mm^2^, 1 ms for ChR2 activation, or 1 s for ArchT activation) around the recorded cell, and postsynaptic responses were recorded in voltage-clamp (for circuit mapping) or current-clamp (for confirmation of ArchT-mediated photinhibition). Functional ChR2 expression was confirmed by recording light-activated action potentials in the target cells (*n* = 3 cells per group, not shown). For testing LH output connections of GAD65_LH_ cells, we chose LH neurons based on their genetic markers (MCH::GFP, orexin::GFP, GAD65::GFP) without noting GAD65 axon location. However, GAD65 axons were dense and abundant everywhere in the LH^16^.

### Chemicals and solutions

For brain slice recordings, ACSF and ice-cold slicing solution were gassed with 95% O_2_ and 5% CO_2_, and contained the following: 125 mM NaCl ACSF, 2.5 mM KCl, 1 mM MgCl_2_, 2 mM CaCl_2_, 1.2 mM NaH_2_PO_4_, 21 mM NaHCO_3_, 2 mM D-(+)-glucose, 0.1 mM Na+-pyruvate, and 0.4 mM ascorbic acid. The slicing solution contained 2.5 mM KCl, 1.3 mM NaH_2_PO·H_2_O, 26.0 mM NaHCO_3_, 213.3 mM sucrose, 10.0 mM D-(+)-glucose, 2.0 mM MgCl_2_, and 2.0 mM CaCl_2_. For standard whole-cell recordings, pipettes were filled with intracellular solution containing the following: 120 mM K-gluconate, 10 mM KCl, 10 mM Hepes, 0.1 mM EGTA, 4 mM K2ATP, 2 mM Na2ATP, 0.3 mM Na2GTP, and 2 mM MgCl_2_ (pH 7.3) with KOH. Gabazine (3 μm) was used where indicated. For in vivo chemogenetic manipulations, CNO was injected i.p. at 0.5 mg/kg body weight in experiments involving hM3Dq. The MCH receptor antagonist SNAP 94847 hydrochloride was injected i.p. at 20 mg/kg body weight (based on ref. ^37^) after being dissolved in distilled water with 10% DMSO and 30 mg/ml (2-Hydroxypropyl)-β-cyclodextrin. All chemicals were from Sigma or Tocris Bioscience.

### Immunohistochemistry

For the immunolabeling of MCH neurons, 50-μm cryosections of pMCH-dependent GCaMP6s injected C57B/l6 mice were stained for MCH with a rabbit antibody to MCH (H-070-47,1:2000, Phoenix Pharmaceuticals) as a primary antibody, and Alexa 555–conjugated donkey antibody to rabbit IgG (A-21244, 1:500, Invitrogen) as a secondary antibody. Slices were then imaged with an Olympus VS120 slide scanner microscope and double labelling of GCaMP with Alexa 555 was quantified with ImageJ.

### Statistical analyses

Statistical tests and descriptive statistics were performed as specified in the figure legends. All experimental animals were included in the analyses (no pre-selection or exclusion). In each experimental dataset at the cellular level, each n was a different cell (no repeated trials from the same cell were used as n values) and cells from at least three mice were analyzed. Before performing parametric tests, data were assessed for normality with a D’Agostino–Pearson omnibus test or Kolmogorov–Smirnov test for small sample sizes. To compare interactions within normally distributed data with repeated measurements, repeated measures ANOVA was used, with multiple comparison tests where appropriate. All statistical tests are two tailed unless otherwise stated. All error bars indicate the standard error of the mean. Analysis was performed with GraphPad Prism and MATLAB (The MathWorks, Inc.).

### Reporting summary

Further information on research design is available in the Nature Research Reporting Summary linked to this article.

## Supplementary information

Supplementary Information

Reporting Summary

## Data Availability

The datasets generated and analyzed during the current study are available from the corresponding author on reasonable request.

## Code availabilty

Custom codes for the acquisition of photometry data (Labview) and for the analysis of photometry data (Matlab) are available from the corresponding author on reasonable request.
