# Supplementary material for: Natural hypothalamic circuit dynamics underlying object memorization
Source: Nat Commun. 2019 Jun 7;10:2505. doi: 10.1038/s41467-019-10484-7 (PMC6555780; doi:10.1038/s41467-019-10484-7)
Supplement: Supplementary file 1 — Supplementary Information [file 41467_2019_10484_MOESM1_ESM.pdf]

**SUPPLEMENTARY FIGURES**

# Natural hypothalamic circuit dynamics underlying object memorization

Kosse and Burdakov

## Supplementary Figure 1

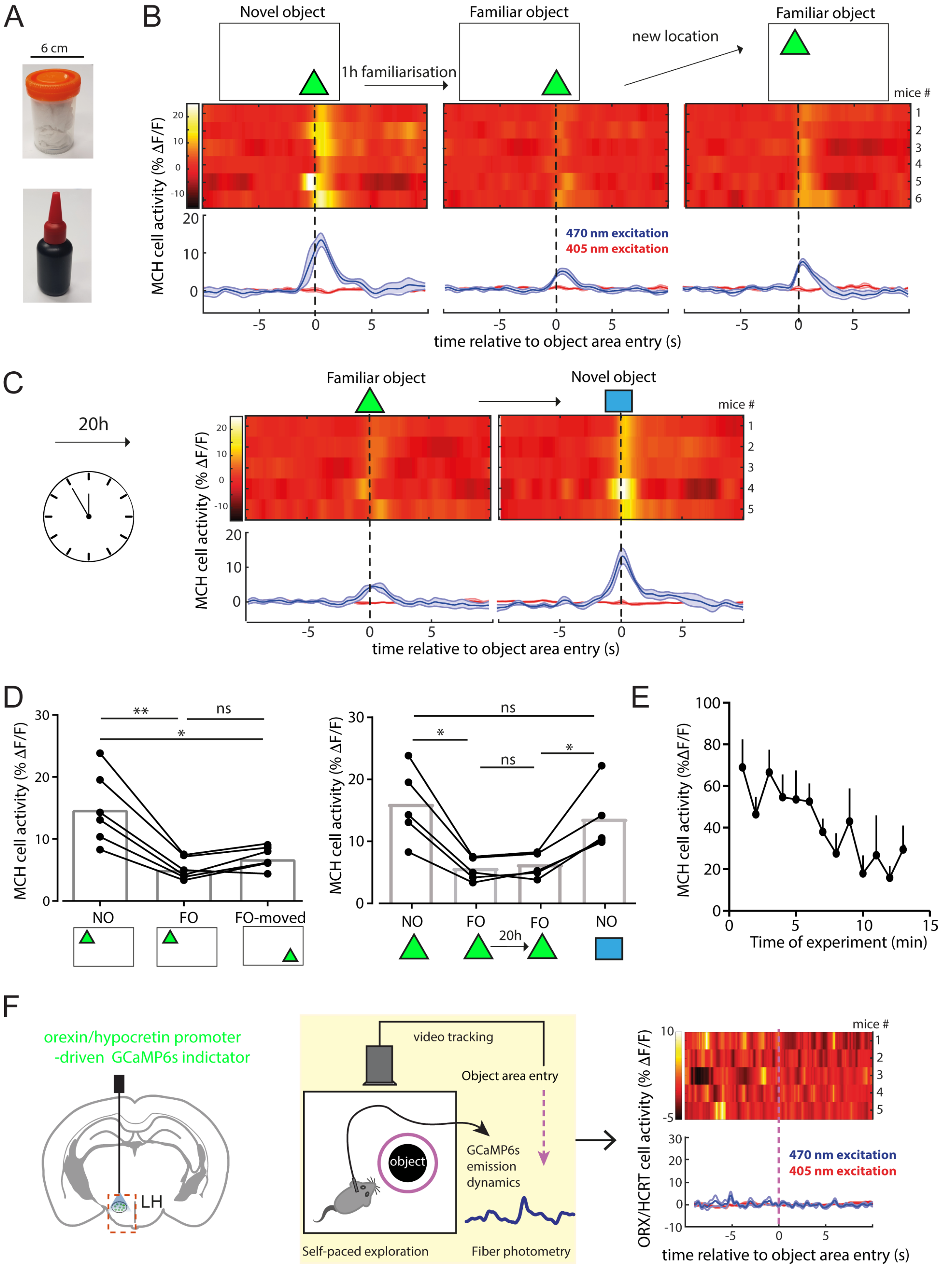


**Specificity of $MCH_{LH}$ cells responses to novel objects: roles of object location, memory retention time, and comparison with other LH cells (A)** Typical examples of objects used in the study. All objects were approximately the same size but differed in shape. **(B-F)** Control data on hypothalamic representations of object encounters. **(B)**, Moving a familiar object to a new location in the cage does not restore large $MCH_{LH}$ cells responses typical of novel objects, indicating that $MCH_{LH}$ cells selectively represent object

novelty rather than object place. Heatmaps of $MCH_{LH}$::GCaMP6s fluorescence aligned to object area entry (each heatmap time-sweep is an average of first 10 entries per mouse; traces below heatmaps are means±s.e.m. of n = 6 mice, also showing negative control fluorescence from 405 nm excitation). **(C)**, Continuation of the experiment shown in B (the experiment was continued in C for 5/6 mice shown in B), showing that the object familiarisation -associated reduction in the $MCH_{LH}$::GCaMP6s signal persists for 20 hours, and can then be reversed by presenting a novel object (n = 5 mice). **(D)**, Left, analysis of group data shown in B: One-way ANOVA $F(1.12, 5.601) = 22.05$, $p = 0.0036$, Tuckey's post test **p = 0.005, *p = 0.0247, ns = p = 0.0863. Right, analysis of group data shown in C: One-way ANOVA $F(1.467, 5.867) = 21.38$, $p = 0.0027$, Tuckey's post test left *p = 0.0186, right *p = 0.0401, ns = p > 0.3… **(E)**, Time-course of novel object area entry-associated MCH cell activity peak size during a typical experiment (means+s.e.m. of n = 5 mice). **(F)** Specificity of MCH cell dynamics: hypocretin/orexin cell activity does not increase during novel object area entry. Left, Calcium indicator targeting (we used a previously validated targeting method characterized in Gonzalez et al, *Current Biology* 2016, 26(18): 2486-2491). Center, Experimental set-up. Right, Hypocretin/$orexin_{LH}$ cell dynamics associated with novel object exploration (data plots are of the type as described above in B, n = 5 mice).
Error bars show s.e.m.

## Supplementary Figure 2

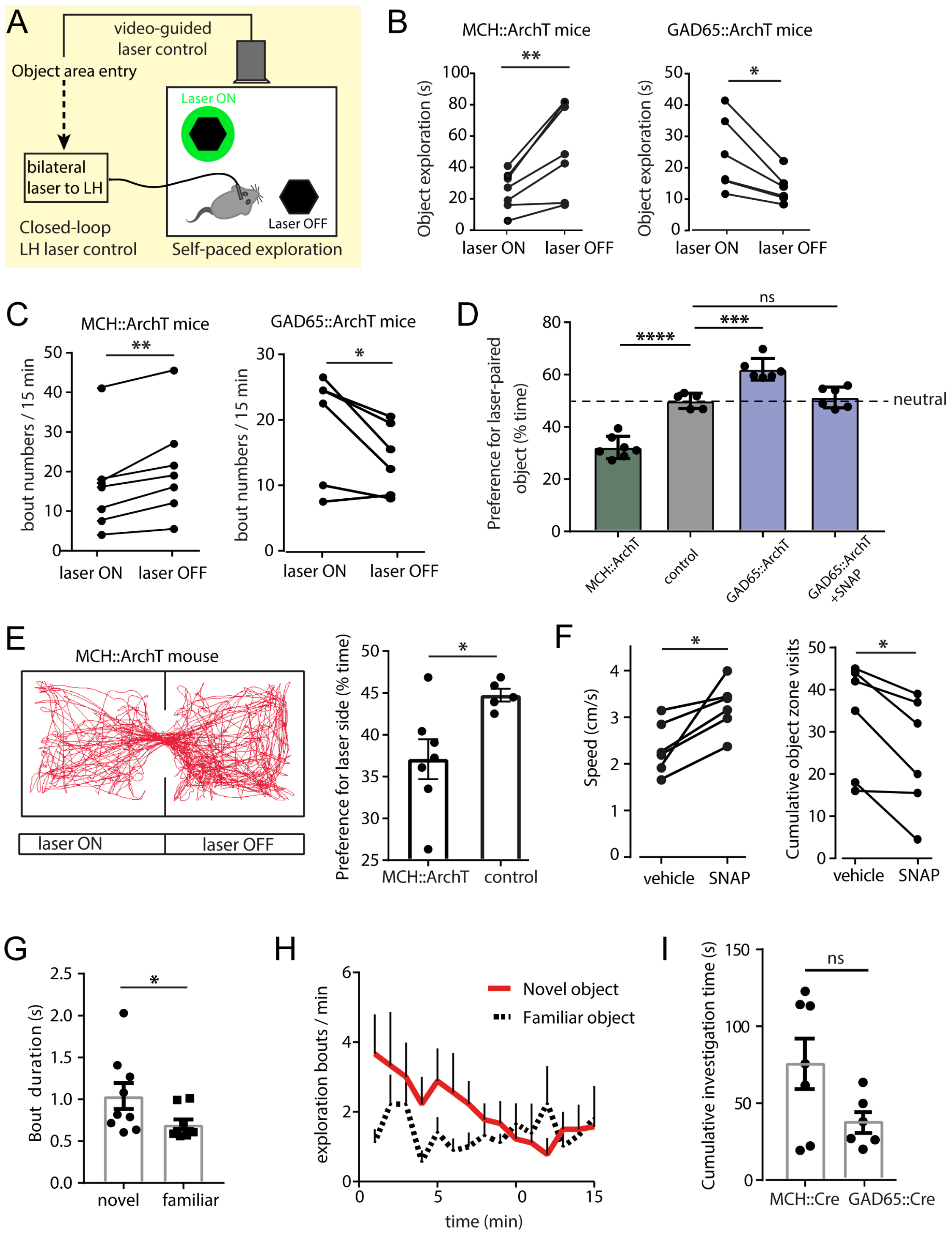


**Control experiments that guided experimental design and interpretation.** (A-D) Control experiments for effect of optogenetic manipulations on object exploration duration (these effects were subsequently controlled for by cumulative exploration time-matching, as described in Methods). Self-paced exploration of two identical novel objects for 10 min with two identical objects placed diagonally while one object and its peri-object space were paired with laser triggering through closed loop-video tracking. Object areas were defined as extending by 3 cm around the object, and automated real-time nose video-tracking (Ethovision XP) scored each time the mouse nose entered the object area as a visit. In experiments with MCH receptor blocker, the MCH antagonists SNAP94847 or vehicle were

injected i.p. 45 min before the experiment started, and familiar objects were familiarised before injection. Objects were assigned according to a crossover design. **(A)** Experimental scheme. **(B)** Quantification of raw data of time spent in peri-object area of the laser ON paired object and control object without laser for MCH::ArchT (left), n = 7 mice, paired t-test: t(6)=3.991, **p= 0.0072 and GAD65::ArchT (right), n=6, paired t-test: t(5)=3.503, *p=0.0172. **(C)**, Quantification of exploration bout numbers at the laser-paired and control peri-object area for MCH::ArchT (left), n = 7 mice, paired t-test: t(6)=4.57,**p=0.0038 and (right) for GAD65::ArchT, n=6, paired t-test: t(5)=2.738, *p= 0.0409. **(D)** Quantification of the relative time spent with the laser-paired peri-object space compared to the overall time of peri-object exploration: One way ANOVA F(3, 20)=65.76, p<0.0001, Dunnett's multiple comparison test: MCH::ArchT (n=7) vs control (MCH::GCaMP) mice (n=5), ****p=0.0001; control (n=5) vs GAD65::ArchT (n=6), ***p=0.002; control (n=5) vs GAD65::ArchT+SNAP (n=6) ns=p=0.9024. **(E)** Real-time place preference (RTPP) effect of $MCH_{LH}$ cell optosilencing. Left, representative trajectory of an MCH::ArchT mouse in a place preference chamber where one side was paired with bilateral LH laser illumination, showing that the mouse spent more time in the laser off side of the chamber. Right, quantification of the preference (time spent) for the laser on side of the chamber for n=7 MCH::ArchT and n=5 control (MCH::GCaMP) mice, unpaired t-test: t(10)=2.607, *p=0.0262. **(F)** Effect of SNAP vs vehicle in LH GAD65::ArchT n=6 mice (left) on speed: paired t-test, t(5)=3.536, *p=0.0166 and (right) on peri-object area visits with two identical novel objects: paired t-test, t(5)=3.731, *p=0.0136. **(G)** Quantification of bout duration (i.e. object area occupancy) during photometry recordings of LH MCH::GCaMP mice (n=9 mice) exploring either novel or familiar objects. Paired t-test: t(8)=2.434, *p=0.0409. **(H)** Quantification of exploration bouts (peri-object area entries) per minute for novel and familiar objects of LH MCH::GCaMP mice (n=9 mice). **(I)** Quantification of overall time spent exploring two identical objects when one is paired with laser illumination for LH GAD65::ArchT (n= 6 mice) and LH MCH::ArchT (n=7 mice), unpaired t-test: t(11)=2.014, ns=p=0.0691.
Error bars show s.e.m. around mean.

## Supplementary Figure 3

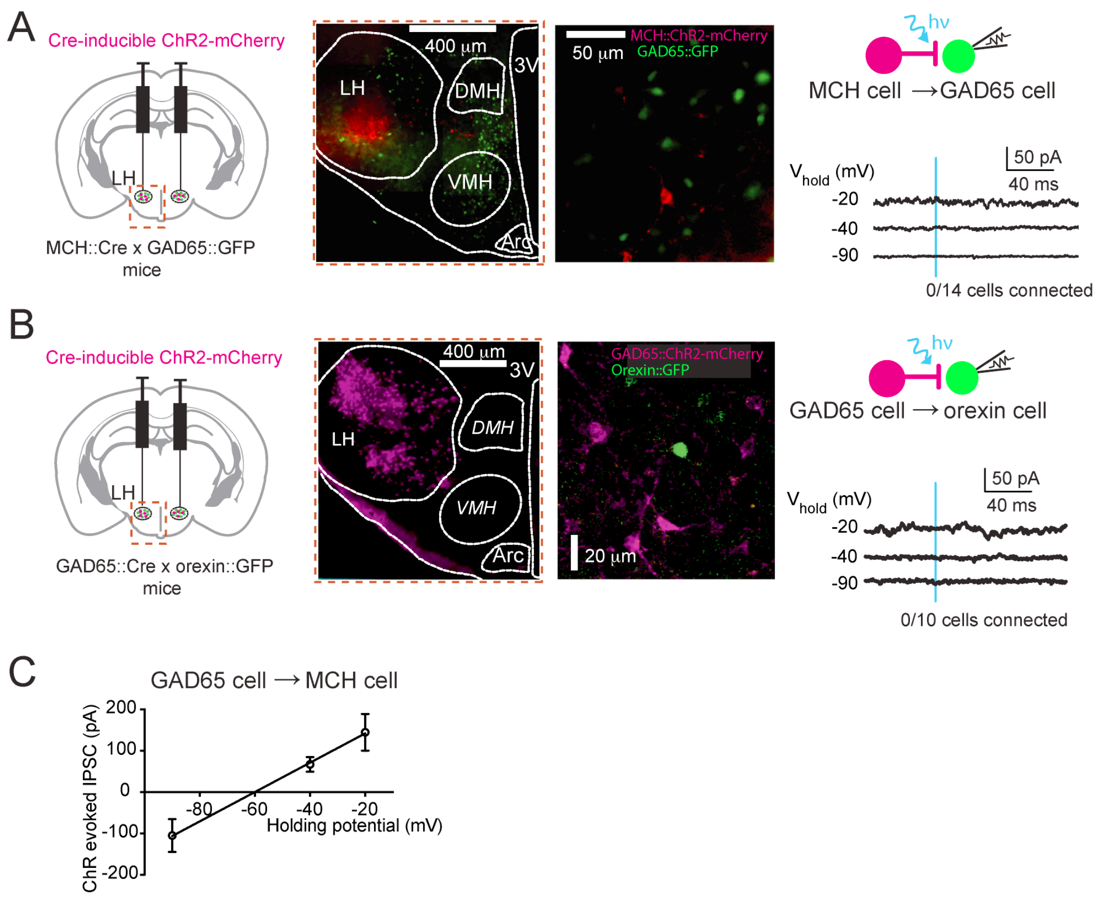


**Additional electrophysiological data on LH local connectivity mapping. (A)** Targeting schematic (left) for expression of ChR2 in $MCH_{LH}$ cells and GFP in $GAD65_{LH}$ cells (middle panels). Right, MCH::$ChR2_{LH}$ optostimulation evokes no currents in GAD65::$GFP_{LH}$ cells (n = 14 cells). **(B)** Targeting schematic (left) for expression of ChR2 in $GAD65_{LH}$ cells and GFP in $orexin_{LH}$ cells (middle panels). Right, GAD65::$ChR2_{LH}$ optostimulation evokes no currents in orexin::$GFP_{LH}$ cells (n = 10 cells). **(C)** Current-voltage relationship of the $GAD65_{LH}$ cell optostimulation-evoked peak inhibitory postsynaptic currents (IPSCs) in $MCH_{LH}$ neurons, at different holding potentials (means±s.e.m. of n=14 cells).

## Supplementary Figure 4

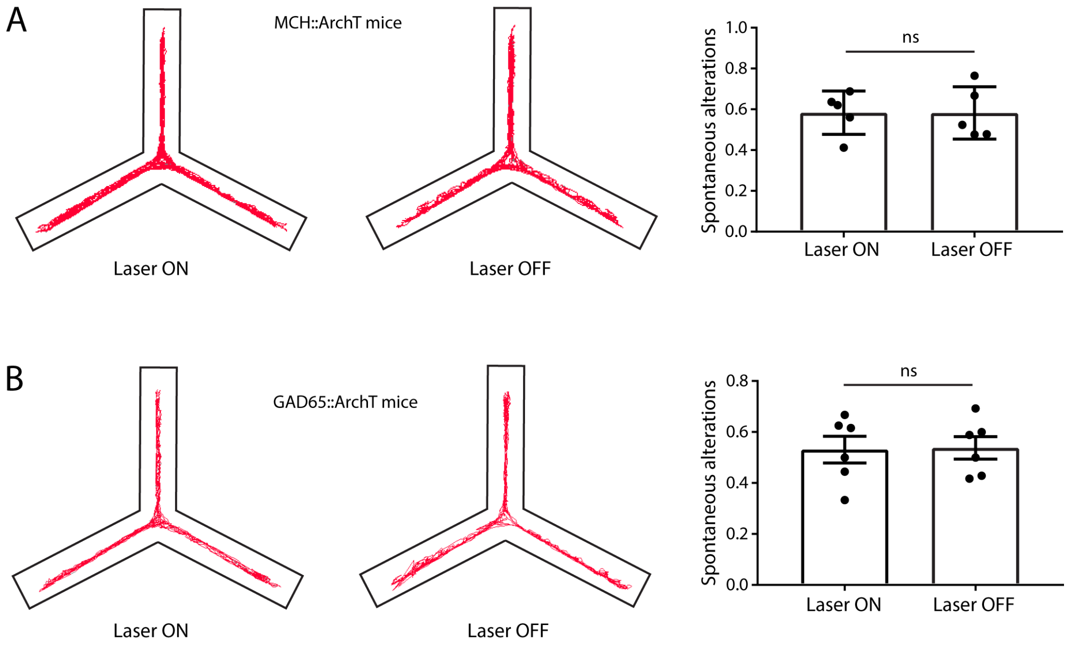


**Spontaneous alterations Y maze data.**
**(A)** Left, representative examples of movement trajectories of an LH MCH::ArchT mouse in a Y-maze during concurrent laser on and off. Right, group data quantification of the proportion of spontaneous alterations (defined as triad of visits to three different arms), n= 5 MCH::ArchT mice, paired t-test: t(4)=0.02115, ns=p=0.9841. **(B)** Same as (A) but with LH Gad65::ArchT mice, n=6 mice, paired t-test: t(5)=0.172, ns=p=0.8702.
Error bars show s.e.m. around mean.

## Supplementary Figure 5

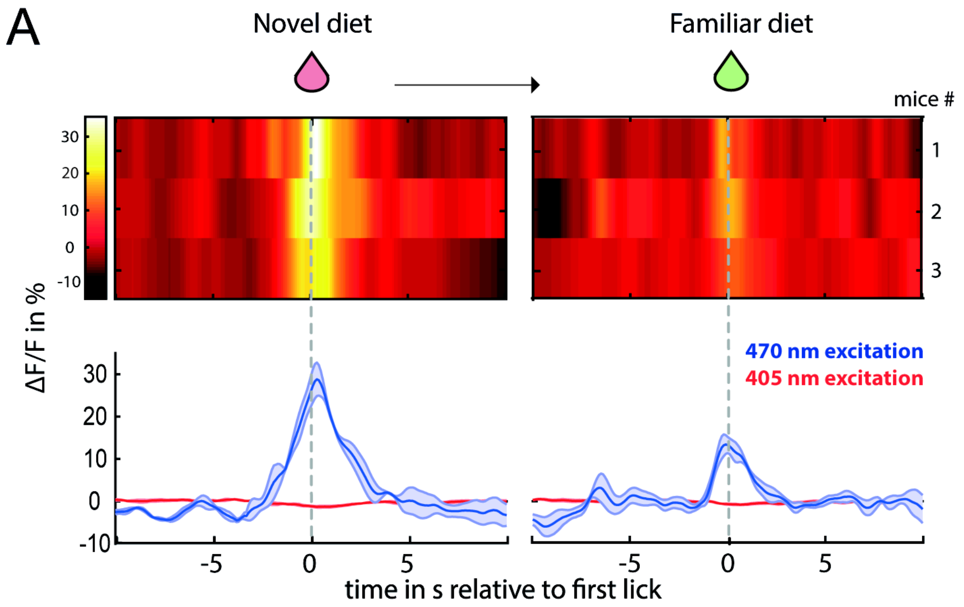


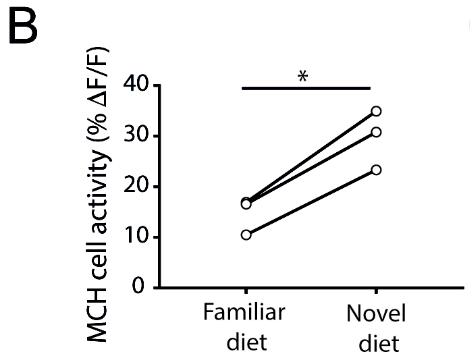


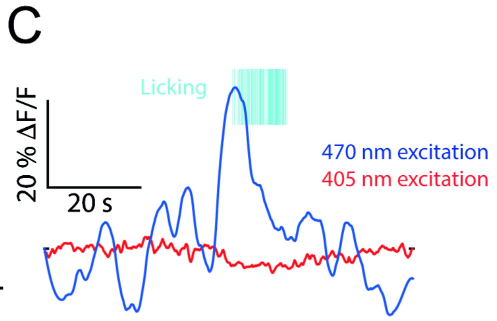


**MCH cell responses to consumption of novel and familiar liquid diet.**
**(A)** Photometry data of n = 3 mice, each an average of recordings aligned to the first lick of the first 3 lick bouts in response to novel and familiar liquid diets (strawberry milkshake or apple juice). Licks were recorded and time-stamped with a lick sensor connected to the food spout (method described in Gonzalez et al, *Current Biology* 2016, 26: 2486-2491). Heatmaps represent the averaged data of one mouse per line, whist the graph below shows an average of the heatmap data (means and s.e.m). **(B)** Quantification of data in **(A)** comparing the peak activity of n = 3 mice, paired t-test: $t(2)=9.727$, *$p=0.0104$. **(C)** Representative example of raw data showing the photometry data during 405nm and 470nm excitation and simultaneous touch sensor recordings of the spout delivering the novel liquid diet.
