# Supplementary material for: Natural hypothalamic circuit dynamics underlying object memorization
Source: Nat Commun. 2019 Jun 7;10:2505. doi: 10.1038/s41467-019-10484-7 (PMC6555780; doi:10.1038/s41467-019-10484-7)
Supplement: Supplementary file 2 — Reporting Summary [file 41467_2019_10484_MOESM2_ESM.pdf]

nature research

Corresponding author(s): Denis Burdakov
Last updated by author(s): 2019/05/16

Reporting Summary

Nature Research wishes to improve the reproducibility of the work that we publish. This form provides structure for consistency and transparency in reporting. For further information on Nature Research policies, see Authors & Referees and the Editorial Policy Checklist.

Statistics

For all statistical analyses, confirm that the following items are present in the figure legend, table legend, main text, or Methods section.

n/a	Confirmed	
☐	☒	The exact sample size (n) for each experimental group/condition, given as a discrete number and unit of measurement
☐	☒	A statement on whether measurements were taken from distinct samples or whether the same sample was measured repeatedly
☐	☒	The statistical test(s) used AND whether they are one- or two-sided<br>*Only common tests should be described solely by name; describe more complex techniques in the Methods section.*
☒	☐	A description of all covariates tested
☐	☒	A description of any assumptions or corrections, such as tests of normality and adjustment for multiple comparisons
☐	☒	A full description of the statistical parameters including central tendency (e.g. means) or other basic estimates (e.g. regression coefficient) AND variation (e.g. standard deviation) or associated estimates of uncertainty (e.g. confidence intervals)
☐	☒	For null hypothesis testing, the test statistic (e.g. F, t, r) with confidence intervals, effect sizes, degrees of freedom and P value noted<br>*Give P values as exact values whenever suitable.*
☒	☐	For Bayesian analysis, information on the choice of priors and Markov chain Monte Carlo settings
☒	☐	For hierarchical and complex designs, identification of the appropriate level for tests and full reporting of outcomes
☒	☐	Estimates of effect sizes (e.g. Cohen's d, Pearson's r), indicating how they were calculated

Our web collection on statistics for biologists contains articles on many of the points above.

Software and code

Policy information about availability of computer code

Data collection	Video tracking: Noldus Ethovision XT 11.5, Photometry and laser triggering: custom labview (2014) code
Data analysis	Matlab R2016a and GraphPad Prism (7)

For manuscripts utilizing custom algorithms or software that are central to the research but not yet described in published literature, software must be made available to editors/reviewers. We strongly encourage code deposition in a community repository (e.g. GitHub). See the Nature Research guidelines for submitting code & software for further information.

Data

Policy information about availability of data

All manuscripts must include a data availability statement. This statement should provide the following information, where applicable:

- Accession codes, unique identifiers, or web links for publicly available datasets
- A list of figures that have associated raw data
- A description of any restrictions on data availability

The datasets generated during and analysed during the current study are available from the corresponding author on reasonable request. Custom codes for the acquisition of photometry data (Labview) and for the analysis of photometry data (Matlab) are available from the corresponding author on reasonable request.

Field-specific reporting

Please select the one below that is the best fit for your research. If you are not sure, read the appropriate sections before making your selection.

☒ Life sciences ☐ Behavioural & social sciences ☐ Ecological, evolutionary & environmental sciences

Life sciences study design

All studies must disclose on these points even when the disclosure is negative.

Sample size	Sample sizes were determined by consulting existing literature using similar techniques and were kept to a minimum to be compliant with local animal usage policies.
Data exclusions	It was pre-established that mice will be excluded that do not show appropriate virus expression.
Replication	For behavioural experiments all reported n come from separate animals, representing a biological replicate. For in vitro experiments data was collected from at least 3 different animals, represent different biological replicates.
Randomization	For most experiments (photometry, optogenetics) experimental mice also served as internal control by either having a 405nm movement control for photometry and having laser on and off treatment for optogenetics (in a latin square like design). Drug treatments and behavioural tests for familiar and nove objects were alternated in a latin square like crossover design. Allocation of objects for familiarization and novel object recognition tasks was allocated in a latin square cross-over design and alternated in sequence for photometry.
Blinding	Behavioural scoring was done automatically via Ethovision to avoid any human bias.

Reporting for specific materials, systems and methods

We require information from authors about some types of materials, experimental systems and methods used in many studies. Here, indicate whether each material, system or method listed is relevant to your study. If you are not sure if a list item applies to your research, read the appropriate section before selecting a response.

Materials & experimental systems

n/a	Involved in the study	
☐	☒	Antibodies
☒	☐	Eukaryotic cell lines
☒	☐	Palaeontology
☐	☒	Animals and other organisms
☒	☐	Human research participants
☒	☐	Clinical data

Methods

n/a	Involved in the study	
☒	☐	ChIP-seq
☒	☐	Flow cytometry
☒	☐	MRI-based neuroimaging

Antibodies

Antibodies used	rabbit antibody to MCH (H-070-47,1:2000, Phoenix Pharmaceuticals) as a primary antibody and Alexa 555–conjugated donkey antibody to rabbit IgG (A-21244, 1:500, Invitrogen) as a secondary antibody
Validation	Both antibodies were validated by the manufacturer and additionally did not show any non-specfic staining outside the hypothalamus.

Animals and other organisms

Policy information about studies involving animals; ARRIVE guidelines recommended for reporting animal research

Laboratory animals	mice, only males for behavior experiments, females and males for in vitro experiments
Wild animals	*Provide details on animals observed in or captured in the field; report species, sex and age where possible. Describe how animals were caught and transported and what happened to captive animals after the study (if killed, explain why and describe method; if released, say where and when) OR state that the study did not involve wild animals.*
Field-collected samples	*For laboratory work with field-collected samples, describe all relevant parameters such as housing, maintenance, temperature, photoperiod and end-of-experiment protocol OR state that the study did not involve samples collected from the field.*
Ethics oversight	All procedures followed United Kingdom Home Office regulations and were approved by the Animal Welfare and Ethical Review Panel of the Francis Crick Institute.

Note that full information on the approval of the study protocol must also be provided in the manuscript.
